# Experimental and Numerical Analysis on the Impact Wear Behavior of TP316H Steel

**DOI:** 10.3390/ma15082881

**Published:** 2022-04-14

**Authors:** Xu-dong Chen, Li-Wen Wang, Qi-hang Yu, Fan Zhang, Kun Mo, Shi-Lin Ming, Zhen-Bing Cai

**Affiliations:** 1 Key Lab of Advanced Technologies of Materials, Tribology Research Institute, Southwest Jiao Tong University, Chengdu 610031, China; cxdong6758@my.swjtu.edu.cn (X.-d.C.); yqh479087274@126.com (Q.-h.Y.); zhangfan@dongfang.com (F.Z.); 2 DEC Academy of Science and Technology Co., Ltd., Chengdu 611731, China; wanglw@dongfang.com (L.-W.W.); mokun@dongfang.com (K.M.); mingshilin@foxmail.com (S.-L.M.)

**Keywords:** TP316H steel, contact-force model, dynamic response, impact wear mechanism

## Abstract

In this work, the contact force model and experiment methods were used to study the dynamic response and impact wear behavior of TP316H steel. The Flore model and the classic Hertz model were selected for comparison with the experimental results, and the model was revised according to the section parameters of the TP316H tube. The results show that there is a large difference between the models without considering the effect of structural stiffness on the impact system and the test results, whereas the revised model has a good agreement. With the rise in impact mass, the coefficient of restitution increases from 0.65 to 0.78, whereas the energy dissipation and wear volume decrease. Spalling, delamination, plastic deformation, and oxidative wear are the main impact wear mechanism of TP316H steel.

## 1. Introduction

The contact-impact occurs in the mechanical system with clearance fits under the influence of external forces. Macroscopically, the impact process is a complex physical phenomenon, accompanied by the characteristics of very short duration, high-contact force, rapid energy dissipation, and large speed changes of the bodies [1]. At the microscopic level (contact interface), repeated impacts cause tribo-chemical reactions between the two bodies, which eventually lead to oxidation, wear, fatigue, and material transfer [2]. The contact-impact is a complex physical and chemical process under actual working conditions (such as high temperature or fluid). The contact-impact and the wear, fatigue, and cracks caused by it have been extensively studied [3,4,5,6,7].

**Figure 1:**
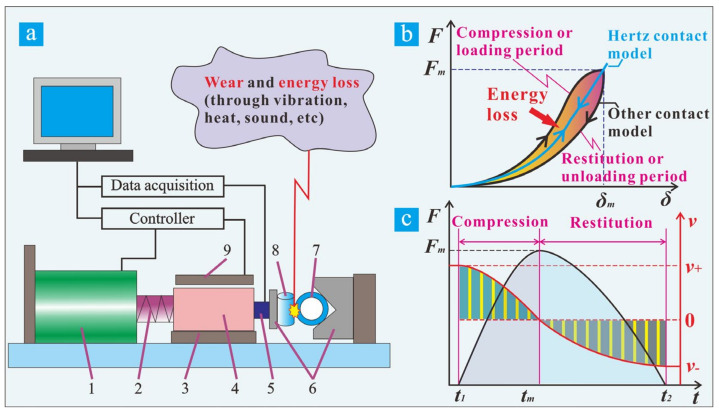
Test equipment (**a**): (1) voice coil motor, (2) damping punch, (3) guide rail, (4) impact block, (5) force sensor, (6) fixture, (7) sample, (8) tri-bro pair, (9) velocity sensor; and the dynamic response of an impact event: (**b**) contact force versus deformation, (**c**) contact force and velocity versus time.

The early contact force model was proposed by Hertz in 1882 [8], which is a completely elastic contact force model and does not consider the energy dissipation during the impact process. Based on Hertz’s contact theory, researchers improved the contact force model by adding either a linear spring connected to a linear damper or a nonlinear viscoelastic element [9,10]. The new models take into account the energy dissipation and have been well researched and developed [11,12,13]. At present, the consensus view is that the contact-impact process can be regarded as two stages (Figure 1b,c [11]: namely the compression, approaching or loading period, and the restitution, separating or unloading period [14,15]. In the compression stage, the contact force and deformation gradually increase from zero to the maximum value. The acceleration and velocity decrease to zero (Figure 1c). In return, the end of the compression stage is the beginning of the restitution stage, the contact force and deformation gradually decrease to zero when the two bodies are separated. Meanwhile, the velocity of the reverse will gradually increase (Figure 1c) [16]. In this process, energy dissipation due to internal damping (through vibration, heat, sound, etc.) usually occurs [17], as shown in Figure 1a,b. It can be defined by Newton’s Law of restitution, that is, the ratio of the velocity after and before the impact, which is also expressed as the coefficient of restitution [18]. The elastic strain energy stored by the material in the compression stage will finally become the kinetic energy of the rebound [19]. Although the theoretical contact force model has been extensively studied, there are few reports on its application to impact wear. This study combines the contact force model and impact wear test to study the dynamic response and wear behavior of the impact interface.

In the heat exchangers of nuclear power, the flow of the heat transfer medium will cause the vibration of the structure, such as the heat transfer tube, against the anti-vibration bar [20]. In the past few decades, scholars have carried out a lot of research on the impact wear of heat transfer tubes, including different contact forms, environments, and materials (Inconel 600, Inconel 690, and 316L) [21,22,23,24]. With the development of nuclear power technology, more and more attention is paid to candidate materials for the fourth generation reactor. The wear properties of 2.25Cr-1Mo steel, a candidate material for heat transfer tubes in a sodium-cooled fast reactor (SFR), are reported [25,26]. Another candidate material for the SFR is TP316H [27], which is used for heat exchangers [28]. There are few studies about the wear of TP316H [29,30]. Thus, this study takes the TP316H steel as the sample and combines existing models and experimental data to develop a revised contact force model applied to the thin-walled tube and discuss its correctness. The wear mechanism and energy dissipation during the impact wear process are discussed. This study also compared the differences between different models and test results, which is not only significant for the development of the contact force model but also provides data support for the engineering practice of TP316H steel.

## 2. Materials and Methods

### 2.1. Materials and Experimental Procedure

In this study, the impact wear of TP316H steel against 316H (the tube support plate material) was carried out under dry conditions. The main component of TP316H steel is Fe, and the mass percentages of other components are C(0.04–0.10), Mn(2.0), P(≤0.04), S(≤0.3), Si(≤0.075), Cr(16.0–18.0), Ni(11.0–14.0), and Mo(2.0–3.0). Table 1 show the mechanical properties of the tribo-pairs [29,30].

The self-developed impact wear test rig with controllable kinetic energy was used for this study, as shown in Figure 1a [31]. It can conveniently realize the impact wear test of different velocities, impact mass, and temperature. The impact velocity and contact force can be recorded in real-time. The energy dissipation and relative deformation between the contact bodies can be analyzed by the kinetic energy theorem and the integral of velocity against time, respectively. A detailed equipment introduction can be referred to in our previous work [31]. Table 2 shows the detailed experimental parameters. The initial impact kinetic energy was 3.5 mJ, the variable was the impact mass, and the impact frequency and the number of cycles were 3 Hz, 10^4^. After the test, scanning electron microscopy (SEM, JSM 7800F, JEOL, Tokyo, Japan), energy-dispersive X-ray spectrometry (EDS, Aztec X-Max 80, Oxford Instrument, Abingdon, UK), and a 3D profiler were used to characterize the morphology, element distribution, and wear volume of the wear scars.

### 2.2. Contact Force Model for Impact Wear

**Figure 2:**
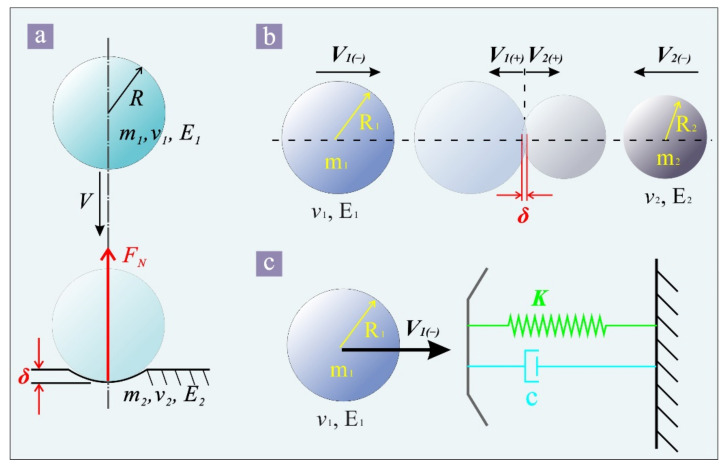
Schematic diagram of impact wear (**a**), idealized contact-impact between two spheres (**b**), and equivalent system (**c**).

P. A. Engel and R. G. Bayer used the Hertz contact force model to analyze the impact wear in 1974 [32]. A ball with a mass of *m*_1_ is assumed to impact a fixed plane, as shown in Figure 2a. The equation of motion for the ball is written in terms of contact force $F_{N}$ and the relative deformation δ [32]:

(1) $$m\ddot{\delta }\;+\;F_{N}\;=\;0,$$

where the $\ddot{\delta }$ is the acceleration of the ball, $F_{N}$ is based on an idealized analysis of the impact of two solid spheres, as shown in Figure 2b, and is expressed as [8]:

(2) $$F_{N}\;=\;K\delta^{n},$$

The *n* is the nonlinear power exponent (usually equal to 3/2). And *K* is the generalized parameter, which depends on the properties of the materials and the radii of the spheres [8]:

(3) $$K\;=\;\frac{4}{3\left(h_{1}\;+\;h_{2}\right)}\;*\;{\left[\frac{R_{1}R_{2}}{R_{1}\;+\;R_{2}}\right]}^{1/2},$$

where *h_l_* is defined for the *l*-th body as [8]:

(4) $$h_{l}\;=\;\frac{1\;-\;v_{l}^{2}}{E_{l}},\;l\;=\;1,\;2.$$

and the variables *v_l_*, *E_l_* is the Poisson’s ratio and Young’s modulus of the two spheres, respectively.

Even though the Hertz contact force model has defects, it was widely used until the 1960s. Then the Kelvin−Voigt model was proposed, taking into account the energy transfer (dissipation) during the impact, and is modeled as a linear spring and a linear damper element, as shown in Figure 2c. The contact force is expressed as [9]:

(5) $$F_{N}\;=\;K\delta \;+\;D\dot{\delta },$$

The right side of the formula represents the elastic force and energy dissipation, where *D* is the hysteresis coefficient and $\dot{\delta }$ is the relative velocity. Although Formula (5) considers energy dissipation, its constitutive law has some weaknesses. At the beginning and end of the impact process, the deformation and the contact force should be null, but the damping component in Formula (5) will make the contact force discontinuous at these two moments. Subsequently, Hunt and Crossley gave a new contact model with a nonlinear viscous-elastic element [10]:

(6) $$F_{N}\;=\;K\delta^{n}\;+\;\chi \delta^{n}\dot{\delta },$$

where the χ is the hysteresis damping factor given by [10]:

(7) $$\chi \;=\;\frac{3K\left(1\;-\;c_{r}\right)}{2{\dot{\delta }}_{\left(-\right)}},$$

where *c_r_* is the coefficient of restitution, and ${\dot{\delta }}_{\left(-\right)}$ is the initial relative velocity. As a result, the expression for this contact force model has the following form [10]:

(8) $$F_{N}\;=\;K\delta^{n}(1\;+\;\frac{3\left(1\;-\;c_{r}\right)}{2}\frac{\dot{\delta }}{{\dot{\delta }}_{\left(-\right)}}),$$

Scholars expanded and developed more models on this basis. Among them, the method of comparing the kinetic energy lost during the impact with the energy obtained by the integration of the contact force on the deformation is widely accepted. The Lankarani−Nikravesh model was proposed in 1989, and its hysteresis damping factor is expressed as [33]:

(9) $$\chi \;=\;\frac{3K\left(1\;-\;c_{r}^{2}\right)}{4{\dot{\delta }}_{\left(-\right)}},$$

The meaning of the variables in Formula (9) is consistent with the previous formula. The contact force can be expressed as [33]:

(10) $$F_{N}\;=\;K\delta^{n}(1\;+\;\frac{3\left(1\;-\;c_{r}^{2}\right)}{4}\frac{\dot{\delta }}{{\dot{\delta }}_{\left(-\right)}}),$$

The Flore model was proposed in 2010, and its hysteresis damping factor is expressed as [17]:

(11) $$\chi \;=\;\frac{8K\left(1\;-\;c_{r}\right)}{5c_{r}\dot{\delta_{\left(-\right)}}},$$

the contact force can be expressed as [17]:

(12) $$F_{N}\;=\;K\delta^{n}(1\;+\;\frac{8\left(1\;-\;c_{r}\right)}{5c_{r}}\frac{\dot{\delta }}{{\dot{\delta }}_{\left(-\right)}}),$$

In recent years, the comparison between various contact force models has been widely reported [1,14,15,17]. For “medium” values of the coefficient of restitution, the Flore model is better at predicting the properties of impact energy dissipation [14]. The Flore model and classic Hertz model were selected to compare with the experimental results in this study.

## 3. Results

### 3.1. Impact Dynamic Response of Thin-Walled Tube

Table 3 lists the experimental results and calculation parameters under varying impact mass. The pre-impact and post-impact velocity are recorded through the experiment, and the generalized parameter (*K*), coefficient of restitution (*c_r_*), and hysteresis damping factor (χ) are calculated. These pre-parameters and Formulas (1) and (12) are written as subroutines of MATLAB and solved.

**Figure 3:**
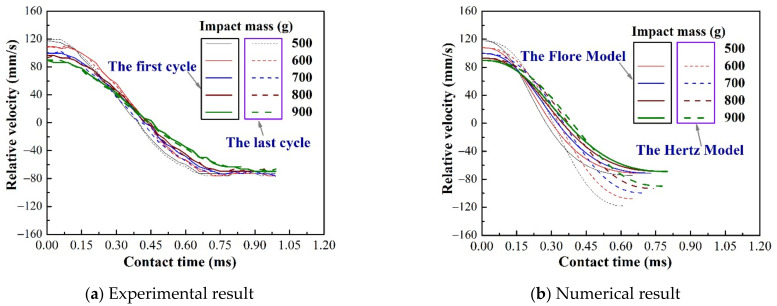
Relative velocity vs time under varying impact mass.

Figure 3 shows the curve of relative velocity versus contact time under varying impact mass. According to the kinetic energy theorem, to keep the initial impact kinetic energy consistent, the pre-impact velocity must decrease as the impact mass increases, as shown in Figure 3a. The results show that post-impact velocity also gradually decreases with the increase in the impact mass, but the magnitude of the decrease is less than the initial impact velocity (Table 3). The results of the Flore model agree with the experimental results because the coefficient of restitution is the same, as shown in Figure 3. The coefficient of restitution of the Hertz model is 1, and its pre-impact velocity is equal to the post-impact velocity, as shown in Figure 3b.

**Figure 4:**
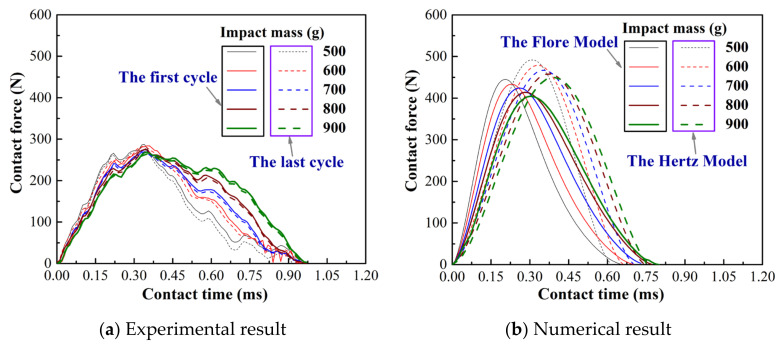
Contact force vs time under varying impact mass.

**Figure 5:**
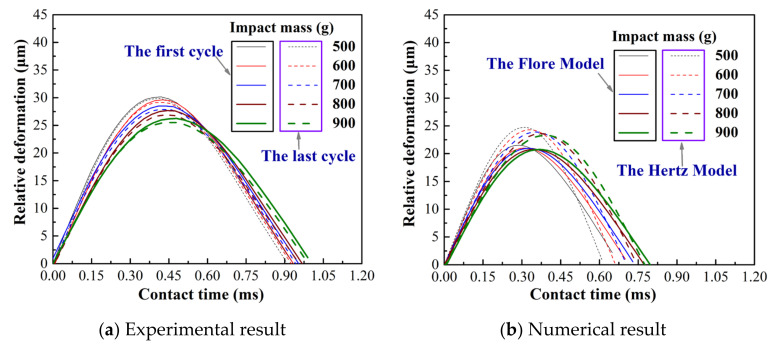
Relative deformation vs time under varying impact mass.

Figure 4 and Figure 5 show the curve of contact force and relative deformation versus contact time under varying impact mass. The experimental results show a slight difference between the first and last impact, as shown in Figure 3a, Figure 4a and Figure 5a. Because the repeated impact process changes the contact area, the accumulation of wear debris simultaneously makes the contact interface change [31]. The numerical results show a significant difference between the Hertz model and Flore model, as shown in Figure 3b, Figure 4b and Figure 5b. This is because the coefficient of restitution causes the Flore model to have smaller results than the Hertz model [1,14,15]. The Flore model has a larger contact force and smaller relative deformation than the experimental results, as shown in Figure 4 and Figure 5. Generally, a structure with higher rigidity is less likely to deform under the same test parameters, and its contact force will also be larger [34].

**Figure 6:**
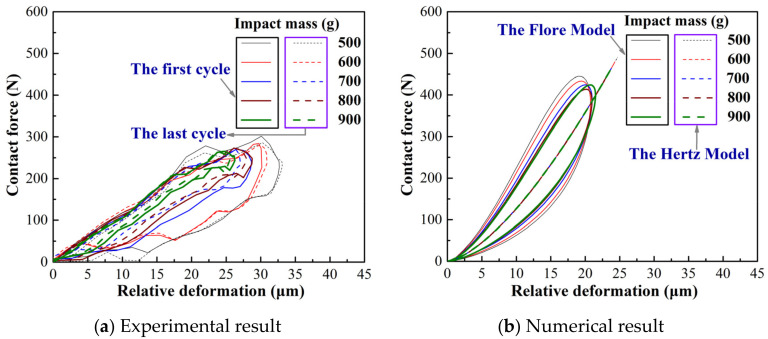
Contact force vs relative deformation under varying impact mass.

The contact force and relative deformation of the Fore model and the experiment all form a closed ring, and its area decreases as the impact mass increases, shown in Figure 6. It represents the work carried out by the damping force, that is, the energy dissipation during the impact [1]. The Hertz model does not take into consideration that the damping force, the contact force-relative deformation curve, is a line. In summary, the Flore model, which considers energy dissipation, fits the experimental results better than the Hertz model. However, there is still a significant difference between the Flore model and the impact wear test.

### 3.2. A Revised Contact Force Model for Thin-Walled Tubes

The current contact force models are developed based on the ideal impact of two solid spheres (Figure 2b). The sample used in this study is a thin-walled TP316H steel tube (wall thickness is 1.2 mm). The rigidity of a solid sphere is greater than that of a thin-walled tube structure of the same material. The dynamic response of the impact interface is not only related to the material but also the structure. A material with a greater hardness [34] or a structure with a greater rigidity (such as a tube with a larger wall thickness or a tube with a smaller span) has a greater contact force and a smaller relative deformation [35,36].

The bending stiffness of a solid cylinder or a tube can be expressed as [37]:

(13) M = EI,

where *E* is Young’s modulus, and *I* is the section second moment of inertia. For a solid cylinder, *I* can be expressed as [37]:

(14) $$I_{1}\;=\;\frac{\pi D^{4}}{4},$$

where *D* is the diameter of the cylinder, and for a tube, *I* can be expressed as [38]:

(15) $$I_{2}\;=\;\frac{\pi (D^{4}\;-\;d^{4})}{4},$$

where *D*, *d* is the outer and inner diameter of the tube.

The bending stiffness of the tube with the same outer diameter is smaller than that of a solid cylinder. A bending stiffness coefficient is introduced to evaluate this difference and is expressed as:

(16) $$\varepsilon \;=\;\frac{M_{2}}{M_{1}}\;=\;\frac{D^{4}\;-\;d^{4}}{D^{4}},$$

ε is a constant and is related to the cross-sectional size of the tube.

The right side of the Flore model can be applied to the tube by multiplying the bending stiffness coefficient, the revised model is expressed as:

(17) $$F_{N}\;=\;\varepsilon K\delta^{n}(1\;+\;\frac{8\left(1\;-\;c_{r}\right)}{5c_{r}}\frac{\dot{\delta }}{{\dot{\delta }}_{\left(-\right)}}),$$

**Figure 7:**
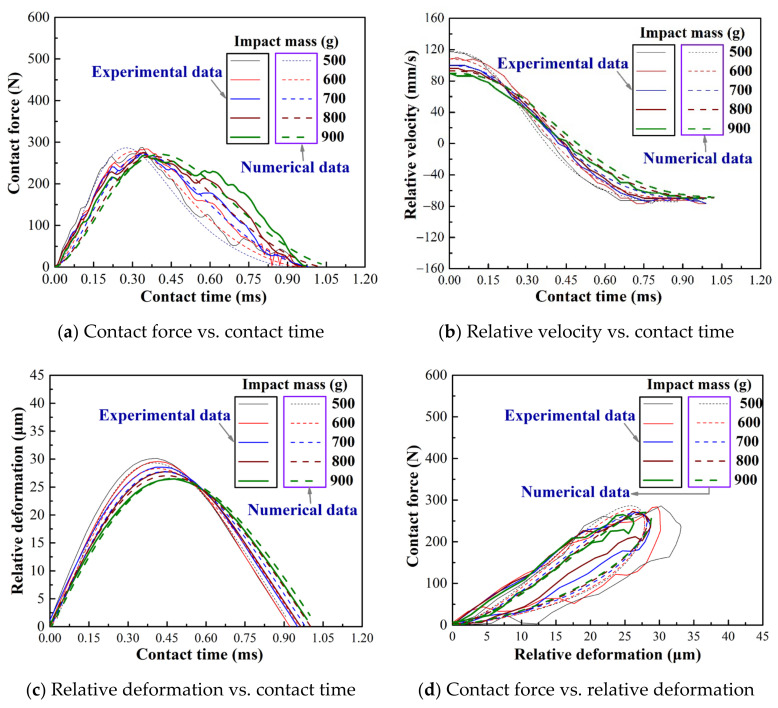
Dynamic response of impact wear with varying impact mass, experimental and revised model results.

Figure 7 shows the comparison between the revised model and the experimental results under varying impact mass. The contact force and relative deformation of the original Flore model are quite different from the experimental results (Figure 4 and Figure 5), whereas the revised model is almost the same as the experimental results, as shown in Figure 7a,c. Meanwhile, the velocity change curve of the revised model is also consistent with the experiment and Flore model. It shows that the revised model makes up the difference and maintains the consistency between the original Flore model and the experiment.

**Figure 8:**
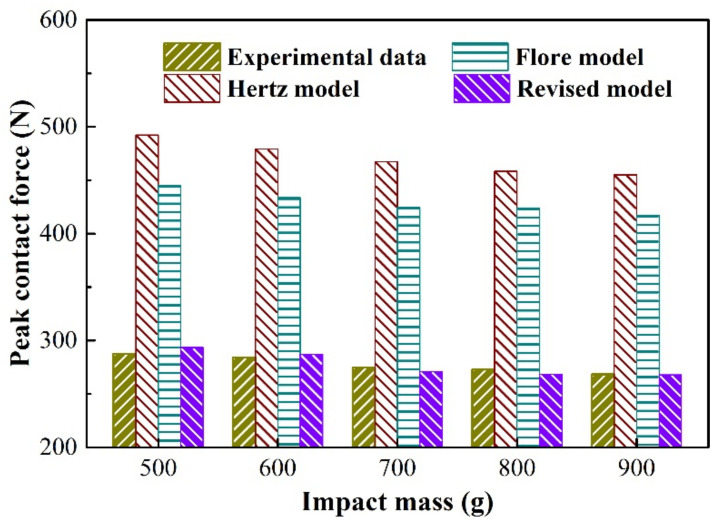
Peak contact force during a contact−impact event with varying impact mass.

**Figure 9:**
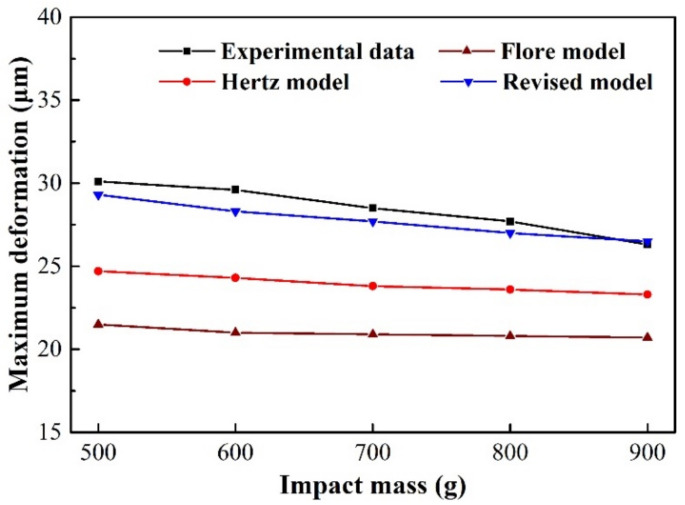
Maximum deformation during a contact-impact event with varying impact mass.

To further analyze the old and revised models and experiment results, Figure 8 and Figure 9 show the two critical parameters in impact wear: peak contact force and maximum relative deformation. The Hertz model that does not consider energy dissipation has the largest peak contact force, and the Flore model that considers energy dissipation is second, as shown in Figure 8. The maximum relative deformation is smallest in the Flore model, followed by the Hertz model, as shown in Figure 9. Since the model does not consider the influence of the structure on the overall stiffness, it has a greater stiffness, that is, a greater ability to resist deformation. Therefore, the contact force of the model is larger than that of the experimental results, and the relative deformation is smaller, as shown in Figure 8 and Figure 9. The revised model is very close to the experiment in terms of peak contact force and maximum relative deformation, as shown in Figure 8 and Figure 9. It shows that the revised model, which considers the structure of the impact body, is more suitable for analyzing the impact wear of thin-walled tubes than the Flore model.

With the same initial kinetic energy, the peak contact force and the maximum relative deformation of the model and test results gradually decrease with the increase in the impact mass, as shown in Figure 8 and Figure 9. The contact time also decreases gradually, as shown in Figure 4, Figure 5 and Figure 7a–c. Inertia is the ability of an object to maintain a state of motion, and the magnitude of inertia is reflected by mass [39]. The increase in mass increases the difficulty of changing its state of motion, and the time for the compression and restitution period also increases. This results in a reduction in the slopes of the force-time and relative deformation-time curves during the compression and restitution periods, as shown in Figure 4, Figure 5 and Figure 7a–c. At the end of the compression period (i.e., exhaustion of the initial kinetic energy), the peak point of the curve decreases as the slope decreases. This is consistent with other reported results [34,39].

### 3.3. Impact Wear Behavior of TP316H

**Figure 10:**
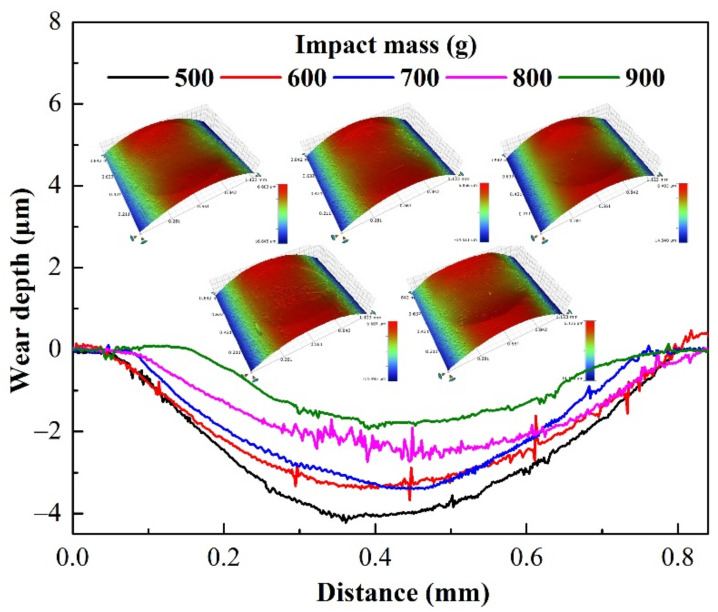
Cross-section profiles and 3D topography of worn scars of varying impact mass.

**Figure 11:**
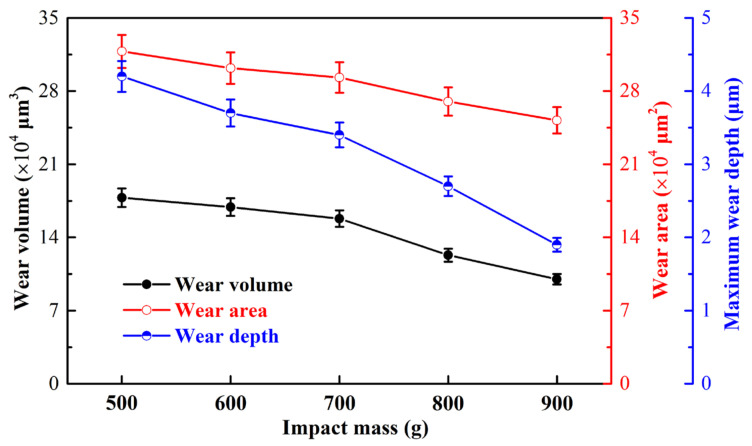
Wear volume, area, and depth of TP316H.

Figure 10 shows the cross-sectional profile and 3D morphology of the wear scar under varying impact mass. Under different impact masses, the wear area is visible and significantly lower than the substrate. The surface of the wear scar is relatively smooth, and the section has some tiny jagged contours (Figure 10), which may be the accumulation of wear debris or the loss of material [35,36]. As the impact mass increases, the wear depth, area, and volume gradually decrease, as shown in Figure 10 and Figure 11.

**Figure 12:**
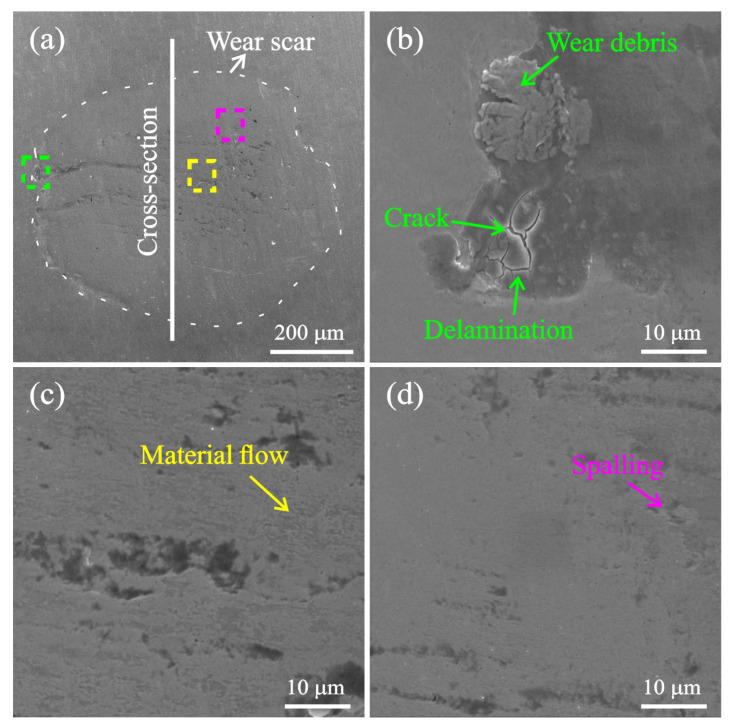
SEM micrographs of wear scar (**a**) and enlarged view (**b**–**d**) under the impact mass of 900 g.

**Figure 13:**
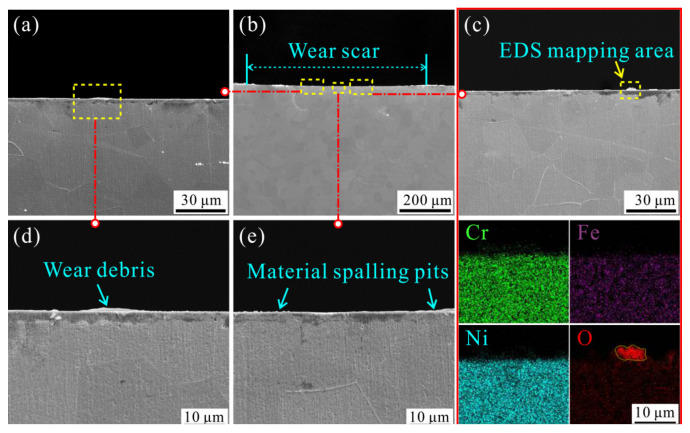
SEM cross-section micrographs of wear scar (**a**–**e**) and EDS (**c**) under the impact mass of 900 g.

Figure 12 shows the SEM images of the wear scar with an impact mass of 900 g. There is an accumulation of wear debris, delamination, and generation of cracks near the boundary of the wear scar, as shown in Figure 12b. Material plastic flow and obvious material spalling pits are also found inside of the wear scar, as shown in Figure 12c,d. The SEM cross-section micrograph of the wear scar, made near the white line in Figure 12a, is shown in Figure 13. The enlarged view of the cross-section clearly shows the accumulated wear debris and some material spalling pits, which are consistent with the cross-sectional profile of Figure 10 and the surface topography of Figure 12. Elemental analysis found that the area where the wear debris accumulated was rich in oxygen, indicating that the impact wear process was accompanied by oxidation of the material [34], as shown in Figure 13c.

## 4. Discussion

### 4.1. The Validity of the Revised Model

The existing contact force model is based on the impact between two solid spheres, and although it considers the effect of the properties of the material, it ignores the effect of structural stiffness on the impact system. The overall stiffness of the two-body impact system is closely related to its structure. The stiffness determines its ability to resist deformation, and the structure with higher stiffness shows a larger peak contact force in the impact test under the same parameters [35,36]. The contact force model is an expression of the contact force, so the most direct way to reduce the contact force is to multiply it by a factor (less than 1). This coefficient must be related to the structure to have broad applicability. Bending stiffness represents the ability of a part to resist bending deformation, and it is a good way to explain the difference between a thin-walled tube and a solid cylinder. As described in Section 3.2, the bending stiffness coefficient is the ratio of the bending stiffness of the thin-walled tube to the cylinder. The Flore model multiplied by the bending stiffness coefficient is in good agreement with the experimental results, as shown in Figure 7.

**Figure 14:**
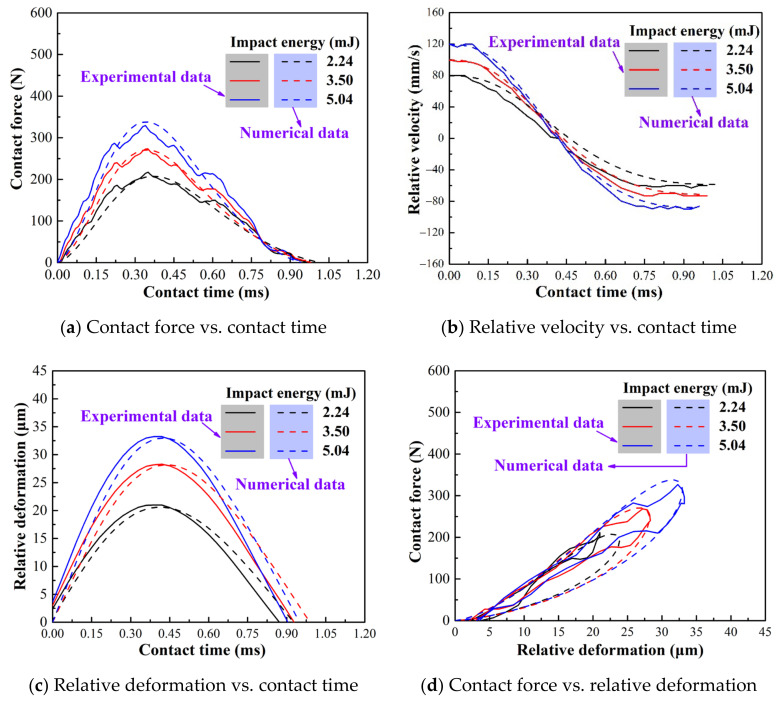
Dynamic response of contact-impact: experimental and numerical results of varied impact energy.

In addition, to further verify the applicability of the revised model, we conducted another set of experiments. The impact mass remains at 700 g and the initial impact kinetic energy changes (2.24, 350, and 5.4 mJ). The experimental results are also in good agreement with the revised model results, as shown in Figure 14. It shows that the revised model has a wide range of validity for the impact wear of thin-walled tubes. The maximum peak contact force and the maximum relative deformation increase with the initial impact kinetic energy, which is consistent with other studies [34,35,36,39].

### 4.2. Energy Dissipation during Impact

The impact wear process is inevitably accompanied by energy dissipation. Evaluating impact wear by calculating the energy loss during impact has been widely used, and the wear volume is proportional to the energy loss [6,35,40]. There are two methods to calculate the energy dissipation, one is to use the kinetic energy theorem, and the formula can be expressed as [22]:

(18) $$\Delta E\;=\;\frac{1}{2}m\left(v_{1}^{2}\;-\;v_{2}^{2}\right),$$

where *m* is the impact mass, *v*_1_*, v*_2_ are the pre-impact and post-impact velocity, respectively.

Another method is to calculate the mechanical work carried out by the damping force, which can be written as [33]:

(19) $$\Delta E\;=\;\oint \chi \delta^{1.5}\dot{\delta }d\delta ,$$

That is the area enclosed by the contact force-relative deformation curve, as shown in Figure 1b.

**Figure 15:**
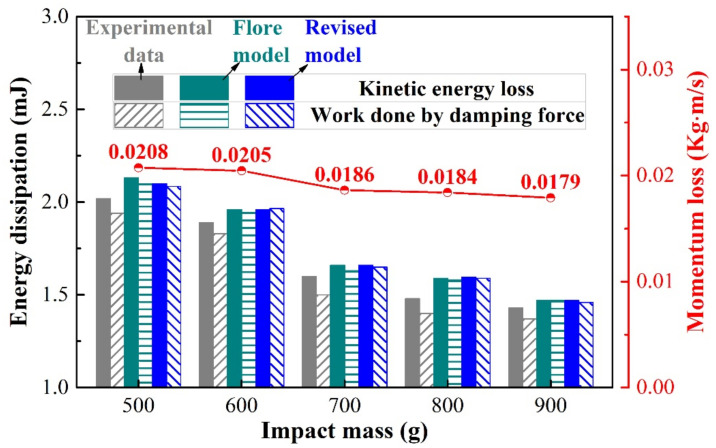
Energy dissipation and momentum loss during a contact-impact event with varying impact mass.

Figure 15 shows the energy dissipation of the experiment and different contact force models under varying impact mass. Since the restitution coefficient in the model comes from the experimental results, the energy dissipation of the revised model, the Flore model, and the experimental results are relatively consistent, and the work carried out by the damping force is almost equal to the kinetic energy dissipation, as shown in Figure 15. This further verifies the correctness of the Flore model and the revised model. As the impact mass increases, the lost energy decreases gradually. Most of the energy lost is used for wear and plastic deformation of material [31,34], and a small part is lost in other forms such as sound and heat [17]. On the one hand, the coefficient of restitution increases with the increase in the impact mass (Table 3), then the kinetic energy loss decreases gradually [14,15]. The increase in mass (inertia) increases the difficulty for the impact block to change its state of motion [39]. When the initial kinetic energy is the same, with the rise in impact mass, the time of the contact force in the compression period becomes longer, and its peak force decreases. Thus, the area enclosed by the contact force and relative deformation will decrease, and the work carried out by the damping force decreases. On the other hand, as the impact mass increases, the momentum loss also decreases, as shown in Figure 15. The loss of momentum represents the product of the contact force and time during the impact process, that is, the impulse [41,42]. As the impact mass increases, the decrease in impulse is also responsible for the decrease in energy loss. In summary, with the rise in impact mass, the energy loss in the impact process decreases, resulting in less energy being used for wear, so the wear volume decreases. This further explains the results in Section 3.3.

## 5. Conclusions

In this study, the dynamic response and wear behavior of the impact interface of thin-walled TP316H steel were studied by combining the contact force model and experiment, and the main conclusions are as follows:

(a) The revised contact force model is in good agreement with the experimental results and is more suitable for studying the dynamic response of the thin-walled tube impact interface than the existing model. This provides a reference for applying the contact force model to impact wear tests.
(b) With the rise in impact mass, the coefficient of restitution increases from 0.65 to 0.78, whereas the peak contact force and maximum relative deformation decrease, and the momentum loss also decreases.
(c) When the kinetic energy is equal, the energy dissipation and wear volume decrease as the impact mass increases. The impact wear mechanism of TP316H steel is mainly spalling, delamination, plastic deformation, and oxidative wear.

## Figures and Tables

**Table 1:** Mechanical properties of tribo-pairs.

Materials	HV_(0.1)_	Poisson’s Ratio	Young’s Modulus	Surface Roughness
TP316H	174.63	0.29	112.98 Gpa	Ra = 0.12 μm
316H	172.55	0.28	89.11 Gpa	Ra = 0.13 μm

**Table 2:** Experimental parameters for the impact wear test.

Impact Wear Test		Sample Geometry	
Tube material	TP316H	Tube	
Rod material	316H	Outer diameter	16 mm
Temperature	Room temperature	Inner diameter	13.6 mm
Impact mass	500 g, 600 g, 700 g, 800 g, 900 g	Length	30 mm
Impact energy	3.50 mJ	Rod	
Impact frequency	3 Hz	Diameter	10 mm
Number of cycles	10^4^	Length	20 mm

**Table 3:** Experimental results and calculation parameters under varying impact mass.

Test Variables	The Experimental Data		The Calculated Data		
	Pre-Impact Velocity (*v*_1_) mm/s	Post-Impact Velocity (*v*_2_) mm/s	*K*	*c_r_*	χ
500 g	117.9	76.4	4.0 × 10^9^	0.65	2.9 × 10^10^
600 g	107.9	73.8		0.68	2.8 × 10^10^
700 g	99.6	73.0		0.73	2.4 × 10^10^
800 g	93.0	70.0		0.75	2.3 × 10^10^
900 g	89.7	69.8		0.78	2.0 × 10^10^

## Data Availability

Not applicable.
